# To Use or Not Use Intraoperative Neuromonitoring: Utilization of Neuromonitoring During Spine Surgeries and Associated Conflicts of Interest, a Cross-Sectional Survey Study

**DOI:** 10.5435/JAAOSGlobal-D-21-00273

**Published:** 2022-03-02

**Authors:** Jesse E. Bible, Madison Goss

**Affiliations:** From the Department of Orthopaedics & Rehabilitation, Penn State Milton S. Hershey Medical Center, Hershey, PA.

## Abstract

There are no universal guidelines that dictate the indications for the use of intraoperative neuromonitoring (IONM) in spine surgery resulting in its variable use. The choice to use IONM has been both cited in malpractice lawsuits and insurance claims, but no data exist regarding surgeons' rationale for making this choice. The goal of this study was to assess (1) the use of certain IONM modalities during common spine surgeries, (2) surgeons' rationale for use of IONM, and (3) IONM practices and potential conflicts of interest associated with its use.

**Methods:**

Respondents were asked to select each IONM modality they used during 20 different surgical scenarios within the spine followed by rating the importance of several reasons when selecting to use IONM. Finally, the occurrence of conflicts of interest, out-of-network billing, and cost were assessed.

**Results:**

Approximately one-half (47%) of respondents who perform anterior cervical diskectomy and fusion/total disk arthroplasty for radiculopathy use IONM, opposed to 76% for myelopathy. The presence of cord compression and/or neurologic symptoms increased IONM use by approximately 30% during trauma cases. Medicolegal was the reason of highest importance when choosing to use IONM (7.4 ± 2.9; mean ± SD), followed by surgeon reassurance (6.2 ± 2.7; *P* < 0.0001 versus medicolegal) and belief it affects patient outcomes (5.2 ± 3.0; *P* = 0.004 versus reassurance).

**Conclusions:**

Although there is increasing use of IONM, this has not translated to an absolute requirement for every spine surgery. Surgeons are faced with opposing influences of the medicolegal system and insurance payers. Future guidelines on using IONM should not be absolute, but rather should consider the risks of each procedure, along with how patients and surgeons value these risks, in addition to the costs. The findings of this study should help to serve as a guide to surgeons, payers, and courts as contemporary, common practices for the use of IONM during spinal surgical scenarios.

Intraoperative neuromonitoring (IONM) use during spine surgeries has become more commonplace as techniques have advanced. However, the clinical benefit and its associated cost are frequently debated.^1,2,3,4^ Moreover, multimodal techniques exist for monitoring different pathways and neural structures.^2^ As such, these is no standard, one-size-fits-all recommended technique for all spine surgeries.

Given the controversial cost-benefit analysis of IONM, many insurance providers consider it not medically necessary or investigational for many spine surgeries.^5,6,7,8,9^ Conversely, courts have either explicitly considered IONM as a legal standard of care or alluded to it constituting a standard of care.^10^ These influences can force a surgeon's hand into underutilization or overutilization of IONM independent of its clinical value.

The addition of IONM can add significant cost to a spine case, thus causing the surgeon, hospital, and payer to further question its value. This has become an especially important consideration within the environment of bundled payments. As other costs associated with spine surgeries become more homogenous and transparent, the fees for similar IONM services can vary significantly, which is especially problematic for patients when billed independent of their hospital charges. This has led to reports of patients getting billed excessive charges for IONM services, or worse, surgeons or hospitals getting back a portion of these charges for using these necessary services.^11^

The purpose of this study is to assess (1) the use of certain IOMN modalities during common spine surgical scenarios, (2) surgeons' rationale for deciding to use IONM, and (3) IONM practices and potential associated conflicts of interest via a survey of spinal surgeons.

## Methods

Institutional review board exemption determination was obtained. A three-part questionnaire was developed to assess IONM use in spine surgery. The first portion asked respondents to select which IONM modalities they routinely use for 20 different surgical scenarios, within cervical, thoracic, and lumbar spine (Table 1). Selection options for each scenario included motor-evoked potentials (MEPs), somatosensory evoked potentials (SSEPs), electromyography (EMG), no neuromonitoring used, and I do not perform this procedure. Respondents were allowed to select more than one option for each scenario.

**Table 1:** Demographic Information for Responding Spine Surgeons

	Respondents (n = 293)
Specialty (%)	
Neurosurgery	60
Orthopaedics	40
Practice (%)	
Private	48
Hospital employed	22
Academic/university	30
Location (%)	
Northeast	30
South	34
Midwest	21
West	15
With tort reform	66
Without tort reform	34
Neuromonitoring model (%)	
External company	79
In-house/internal (tech and neurologist)	21

The second part asked the respondents to individually rate their importance that five different reasons play for a surgeon when deciding to use an IONM modality. Responses were collected quantitatively using a Likert scale where 1 corresponded to low importance and 10 to high importance. The five reasons assessed included (1) belief it affects patient outcomes, (2) medicolegal, (3) required by the hospital, (4) patient expectation, and (5) surgeon reassurance. The third part collected information on the respondents' IONM practice model, known costs associated with its use, conflicts of interest between IONM companies and surgeons and/or hospitals. In addition, the respondents were asked if they felt that IONM companies in their region were frequently billing of out of network for their services. Finally, demographic information was collected for each responding surgeon: specialty, practice setting, and state.

The questionnaire used an online platform for ease of distribution and completion. Initially, it was circulated and piloted to a group of spine surgeons to refine the questions, answer choices, and format to ensure clarity and maximize its utility. The online survey link was then circulated via Cervical Spine Research Society newsletter and e-mail addresses. E-mail addresses were obtained by using online member directories, institutional/hospital/health system/practice websites, personnel websites, Doximity, and PubMed.

Result analyses mainly focused on descriptive analyses, given the primary goals of the survey study. SPSS 25.0 software was used for further statistical analyses. Dichotomous data were compared using Fisher exact tests, whereas Mann-Whitney *U* and Kruskal-Wallis tests were used for comparisons of nonparametric continuous data.

## Results

The survey link was sent to the published e-mail addresses of 1101 surgeons. The online survey was started by 196 surgeons. Of which, 193 of them completed it, leading to a completion rate of 98.5% and a response rate of 17.5%. Sixty percent of respondents were neurosurgeons, whereas 40% were orthopaedic surgeons (Table 1). Thirty percent of surgeons reported practicing within an academic/university setting.

### Surgical Scenarios

#### Radiculopathy Versus Myelopathy

Only 47% of surgeons who perform anterior cervical diskectomy and fusion/total disk arthroplasty (ACDF/TDA) for radiculopathy reported using IONM, whereas IONM use increased to 76% during ACDF/TDA for myelopathy (Tables 2–4). Similarly, IOMN is used 54% of the time for corpectomies performed for radiculopathy, as opposed to 82% for myelopathy. This still leaves 18% to 27% of surgeons performing the surveyed surgeries for myelopathy not using any IONM (24% ACDF/TDA, 18% corpectomy, 27% cervical laminectomy/laminoplasty, and 27% thoracic laminectomy).

**Table 2:** Cervical Spine Surgical Scenarios

Surgical Scenario	Percent of Spine Surgeons				
	Do Not Perform the Procedure	No Monitoring	EMG	SSEPs	MEPs
ACDF/TDA for radiculopathy (R) or myelopathy (M)	R: 2 M: 1	R: 53 M: 24	R: 40 M: 57	R: 45 M: 75	R: 35 M: 66
Corpectomy for radiculopathy (R) or myelopathy (M)	R: 7 M: 2	R: 46 M: 18	R: 43 M: 59	R: 52 M: 80	R: 42 M: 72
Laminoforaminotomy for radiculopathy	4	65	31	32	21
Laminectomy and fusion/laminoplasty for myelopathy	3	27	57	71	60
Deformity with or without osteotomies	17	13	66	86	80
Posterior instrumented fusion for pseudarthrosis	6	46	43	53	39
Posterior instrumented fusion for trauma without cord compression or neurologic symptoms	8	37	47	62	51
Posterior instrumented fusion for trauma with cord compression, but without neurologic symptoms	8	21	60	80	72
Posterior instrumented fusion for trauma with neurologic symptoms	8	19	61	80	73

ACDF/TDA = anterior cervical diskectomy and fusion/total diskarthroplasty, EMG = electromyography, MEP = motor-evoked potential, SSEP = somatosensory evoked potential

**Table 3:** Thoracic Spine Surgical Scenarios

Surgical Scenario	Percent of Spine Surgeons				
	Do Not Perform the Procedure	No Monitoring	EMG	SSEPs	MEPs
Laminectomy for myelopathy	5	27	45	72	63
Thoracolumbar deformity with or without osteotomies	18	10	60	89	77
Posterior instrumented fusion for trauma without cord compression or neurologic symptoms	13	34	45	65	54
Posterior instrumented fusion for trauma with cord compression, but without neurologic symptoms	13	20	52	80	73
Posterior instrumented fusion for trauma with neurologic symptoms	13	18	53	81	75

EMG = electromyography, MEP = motor-evoked potential, SSEP = somatosensory evoked potential

**Table 4:** Lumbar Spine Surgical Scenarios

Surgical Scenario	Percent of Spine Surgeons				
	Do Not Perform the Procedure	No Monitoring	EMG	SSEPs	MEPs
Laminectomy	2	83	16	14	5
Laminectomy with posterior instrumented fusion	3	46	51	40	17
ALIF	13	64	31	29	8
Lateral lumbar interbody fusion (XLIF/OLIF/DLIF)	21	19	77	47	23

EMG = electromyography, MEP = motor-evoked potential, SSEP = somatosensory evoked potential

#### Deformity

Of the surgeons performing deformity surgeries, 87% use IONM during cervical deformity cases and 90% during thoracolumbar deformity cases.

#### Trauma

Use of IONM was reported by 63% of surgeons during posterior cervical surgeries for trauma without cord compression or neurologic symptoms. This use increased to 79% with the additional of cord compression without neurologic symptoms (*P* = 0.001) and 81% with neurologic symptoms present (*P* = 0.784). Similar findings were seen for thoracolumbar trauma scenarios: 66% IONM use with no cord compression or neurologic symptoms, 80% with cord compression without symptoms (*P* = 0.002), and 82% with symptoms (*P* = 0.887).

#### Lumbar

Use of some form of IONM was reported by 17% of surgeons during isolated lumbar laminectomy procedures. The addition of a posterior instrumented fusion increased use to 54%, mainly in the form of SSEPs and EMG.

#### Multimodal Intraoperative Neuromonitoring at the Spinal Cord Level

During all cervical and thoracic scenarios when SSEPs are used, 14% of the time concurrent MEPs are not used. For cervical radiculopathy scenarios (ACDF/corpectomy/laminoforaminotomy) when SSEPs are used, 24% of the time concurrent MEPs are not used. For myelopathy, deformity, cord compression, and neurologic symptom scenarios when SSEPs are used, 11% of the time concurrent MEPs are not used.

Concurrent EMG is used with SSEPs during 74% of time for all cord level cases, 87% for radiculopathy cases, and 64% for myelopathy/deformity/cord compression/neurological symptom scenarios.

#### Limited-Use Intraoperative Neuromonitoring

Only 2% of respondents denied use of IONM for any of surveyed surgical scenarios (4% of neurosurgeons and 0% of orthopaedic surgeons). Two percent limit use of IONM to only deformity cases, whereas 2% use IONM for only lateral lumbar interbody cases. Finally, 11% of respondents only used IONM for deformity, myelopathy, trauma case with cord symptoms, or lateral lumbar fusion scenarios. We found no significant differences between those from states with and without tort reform in regard to no or limited use of IONM.

### Reasoning for Using Neuromonitoring

**Figure 1:**
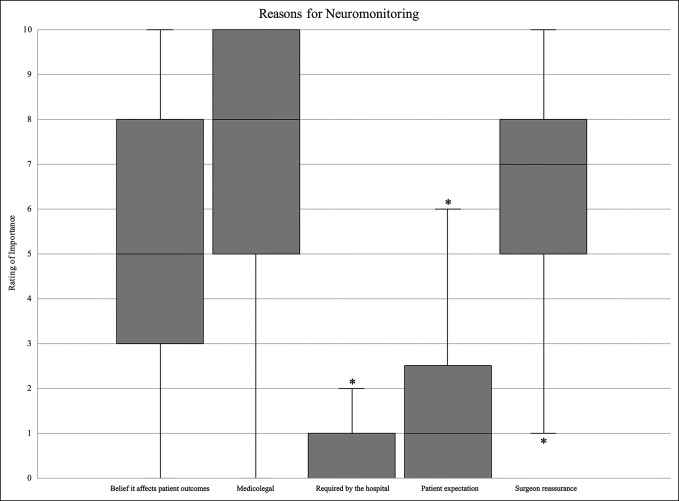
Level of importance for each of five surveyed reasons for neuromonitoring shown as boxplots. Statistically significant differences were seen between medicolegal and surgeon reassurance (*P* < 0.0001), medicolegal and belief it affects outcomes (*P* < 0.0001), and surgeon reassurance and belief it affects patient outcomes (*P* = 0.004). *<0.05. ALIF = anterior lumbar interbody fusion, XLIF = extreme lumbar interbody fusion, OLIF = oblique lumbar interbody fusion, DLIF = direct lateral interbody fusion.

Medicolegal was the reason of highest importance for surgeons choose to use IONM (7.4 ± 2.9; mean ± SD) (Figure 1). This was significantly higher than the next important reason, surgeon reassurance (6.2 ± 2.7; *P* < 0.0001). Next was the belief that it affects patient outcomes (5.2 ± 3.0; *P* = 0.004 compared with surgeon assurance). Patient expectation and hospital requirements were rated of low importance (1.9 ± 2.3 and 1.1 ± 1.5, respectively).

### Conflicts of Interest and Out-of-Network Billing

Known conflicts of interest associated with IONM use within their geographical region were reported by 27% of surgeons, with the most common conflict being between surgeons and monitoring companies (23%) (Table 5). Out-of-network billing was felt to be occurring frequently by 54% of surveyed surgeons. Only 36% of surgeons were aware of the cost associated with IONM use, with 28% of these surgeons reporting a cost of at least $5000.

**Table 5:** Conflicts of Interest, Out of Network Billing, and Cost Associated with Intraoperative Neuromonitoring during Spine Surgery

Question	Percent of All Respondents	Percent of Respondents Using External Monitoring Companies (79%)
Are you aware of any conflicts of interest within your geographical region?	20	25
Hospital versus neuromonitoring company	12	14
Surgeons versus neuromonitoring company	23	28
Do you feel neuromonitoring companies are frequently billing out of network?	54	50
Do you know how much neuromonitoring adds to the cost of the case?	34	38
$0-5,000	72 (of 34)	73 (of 38)
$5,000-15,000	25 (of 34)	23 (of 38)
$15,001-50,000	3 (of 34)	4 (of 38)

## Discussion

The use of IONM during spinal surgeries has grown considerably over the last two decades. Compared with Magit et al^12^ study of 139 spine surgeons in 2004, our respondents used various IONM modalities approximately 25% to 50% more frequently. Some of this increase is likely due to increased availability of certain modalities, as only 41% of respondents in the 2004 study had MEPs available. However, another potential reason for heightened use is increasing medicolegal pressure. Surgeons within our study placed medicolegal of highest importance in their reasoning to choose to use IONM, significantly above their belief that it affected patient outcomes.

There remains considerable debate and controversy regarding the utility of IONM. Many professional societies acknowledge the usefulness of IONM during spine surgeries, but stop short of proclaiming IONM techniques as a standard of care.^3,4,10,13,14^ It should be noted that IONM is the only method available to monitor spinal cord function under general anesthesia. Therefore, one could argue that IONM is a benchmark opposed to standard of care.

IONM is not without drawbacks, including cost, variable sensitivity and specificity, and questionable preventive/therapeutic utility. These drawbacks likely contribute to some surgeons' decision to not use IONM as all, whereas most others selectively choose modalities based on each clinical scenario. The presence of myelopathy increased IONM use by 29% compared with radiculopathy for anterior cervical procedures. While the presence of cord compression and/or neurologic symptoms, increased use by approximately 30% during trauma cases at the cord level. However, cord symptoms (myelopathy or traumatic) did not lead to definite use of IONM, as 18% to 27% of surgeons do not use for myelopathy cases and 18% to 19% do not use during cases with traumatic cord symptoms.

The failure to use IONM during spine surgery has been used to fulfill a plaintiff's first requirement in medial malpractice case lawsuit, establishing the requisite standard of care.^10^ In cases Kingsley, Nissen, and Vaccaro,^15-17^ the courts have either explicitly considered IONM as legal standard of care or alluded to it constituting a standard or care. The courts could establish the applicable standard of care, given that IONM is readily available, used by many surgeons, and its use is recommended by multiple professional societies. Many society recommendations or guidelines explicitly state that they are not a medicolegal document and allow for discretionary use of IONM based on the clinical scenario and surgeon judgment. They further state that failure to completely meet some aspects of these recommendations or guidelines cannot be construed to imply negligence or breach of duty. However, prior precedence has been set with other comparable advancing diagnostic technology. In Washington v. Washington Hospital Center, the court, in determining whether anesthesia carbon dioxide monitors should be a standard of care, looked to guidelines that only encouraged, but did not require the use of such monitors.^18^ The court determined that because other hospitals at the time used carbon dioxide monitors and their use was recommended, the plaintiff established the applicable standard of care.^10^

Given that IONM use does not come as an all or none package, an inference of negligence can arise due to failure to use the proper modality or combination of modalities. Compared with decades earlier, MEPs are more readily available and more frequently used. This increase is not without debate due to concern of being overly sensitive and not specific compared with traditional SSEPs. It has been well established that MEPs allow for monitoring of certain neural pathways in the spinal cord that are left uncovered by SSEPs, exemplifying the potential of multimodal techniques for IONM of the spinal cord. However, we found that during all cervical and thoracic scenarios when SSEPs were used, 14% of the time concurrent MEPs are not used. The reasoning behind this limited use remains unknown, but could be due to a surgeon's lack of belief in MEPs efficacy, inconvenience with anesthesia and surgical exposure, and additional cost.

In opposition to the legal pressures that seemingly argue for more universal use of IONM during all spine surgeries, insurance payers do not provide coverage for IONM during all spine surgeries. Most consider the use of IONM modalities during various spine surgeries not medically necessary or investigational, as justification for their lack of coverage.^5,6,7,8,9^ Based on published coverage for IONM from major payers in the United States,^5,6,7,8,9^ 16% to 52% of surveyed surgeons routinely used IONM techniques for surgical scenarios not typically covered by payers. This can potentially put a surgeon in a position to argue for the use of certain technology due to their concern of medicolegal necessity as opposed to their belief of affecting patient outcome.

Some of the recent interest in payers to establish well-defined criteria for IONM coverage may stem from the current lack of uniformity with regard to IONM use and associated billing, leading to more scrutiny from such payers. It is not an infrequent occurrence for patients to first become aware of IONM when they receive a bill from a neuromonitoring company outlining steep charges for out-of-network fees.^11^ Over half of our respondents felt that out-of-network billing was occurring frequently. For many surgeons and patients, IONM services that are out of network might be the only option. Potential contributors to the aforementioned scenario may include insurance companies limiting the number of in-network neurologists, IONM companies' refusal to negotiate lower rates in a limited supply environment, and hospitals not wanting to spend the overhead to negotiate a charge that is frequently sent directly to the patient.^14^

An additional motive for payers to have heightened concern regarding unnecessary use of IONM is the fact that some groups charge excessive fees to gain business by paying the money back to surgeons or hospitals. Over one-quarter of our respondents were aware of conflicts of interest associated with IONM use within their region. Although the practice of kickbacks remains legal in a few states, both the American Medical Association (see detailed opinion statement within references) and the American Society of Neurophysiological Monitoring do not condone the practice, irrespective of patients giving informed consent, or it being considered legal in any state or jurisdiction.^19,20,21,22,23^

This study does have several limitations. It is a cross-sectional survey of spine surgeons who were willing to complete the online survey, thus introducing sampling bias. The survey did not ask about the availability of IONM services, assuming that each surveyed IONM modality was readily available for routine use to each surveyed surgeon. The list of surgical scenarios is not all inclusive. Furthermore, the surveyed scenarios could not account for the specimen of symptomology or pathology within each scenario such as myelopathy or traumatic instability. Finally, lateral lumbar fusion techniques were not separated, thus potentially limiting interpretation of these results.

Although there is increasing use of IONM, this has not translated into an absolute requirement for every spine surgery or even every spine surgeon. Surgeons are faced with opposing influences when deciding whether to use IONM. The medicolegal system views IONM use as an all or none requirement with a growing pressure to treat it as a legal standard of care. This is opposed to insurance payers' interests in limiting its use due to cost, debated benefit, and potential misuse. The current lack of regulation and standardized use is potentially leading to high rates of out-of-network billing and kickbacks to surgeons and hospitals, despite ethical concerns. Future guidelines should not be absolute, but rather should allow for consideration of the risks of each procedure, along with how patients and surgeons value these risks, in addition to costs of not using IONM during various spine surgeries. The findings of this study should help to serve as a guide to surgeons, payers, and courts as contemporary, common practices for the use of IONM during spinal surgical scenarios.
